# Targeted social marketing of PrEP and the stigmatization of black sexual minority men

**DOI:** 10.1371/journal.pone.0285329

**Published:** 2023-05-11

**Authors:** Sarah K. Calabrese, David A. Kalwicz, John F. Dovidio, Sharanya Rao, Djordje X. Modrakovic, Cheriko A. Boone, Manya Magnus, Michael Kharfen, Viraj V. Patel, Maria Cecilia Zea

**Affiliations:** 1 Department of Psychological and Brain Sciences, George Washington University, Washington, DC, United States of America; 2 Department of Prevention and Community Health, George Washington University, Washington, DC, United States of America; 3 Department of Psychology, Yale University, New Haven, CT, United States of America; 4 TAG Treatment Action Group Inc., New York, NY, United States of America; 5 Department of Epidemiology, George Washington University, Washington, DC, United States of America; 6 HIV/AIDS, Hepatitis, STD & TB Administration, DC Department of Health, Washington, DC, United States of America; 7 Department of Medicine, Albert Einstein College of Medicine, Montefiore Health System, Bronx, NY, United States of America; University of Technology Sydney, AUSTRALIA

## Abstract

Disparities in HIV incidence and PrEP uptake suggest a need to prioritize Black sexual minority men (SMM) in PrEP social marketing initiatives. However, images linking Black SMM to HIV and PrEP may inadvertently reinforce stigma. We examined HIV-negative/status-unknown Black SMM’s responses to targeted PrEP advertisements using mixed methods, including an experiment embedded in a longitudinal online survey (Time 1: *n* = 96; Time 2 [eight weeks]: *n* = 73) and four focus groups (*n* = 18). The full factorial experiment included between-groups and within-subjects comparisons. For between-groups comparisons, each participant was randomly assigned to view one of 12 advertisements, which varied by couple composition (Black SMM couple/Black heterosexual couple/multiple diverse couples/no couples) and campaign (*PrEPare for the Possibilities/PlaySure/PrEP4Love*). We examined couple composition, campaign, and interaction effects on: advertisement judgments (Time 1), PrEP stigma (Time 1), PrEP motivation (Times 1 and 2), and PrEP behavior (Time 2). For within-subjects comparisons, each participant viewed all 12 advertisements, and we examined couple composition, campaign, and interaction effects on advertisement judgments (Time 2). Focus group participants discussed advertising preferences and responded to the same set of advertisements. For between-groups and within-subjects comparisons, we found significant couple composition effects but no or limited campaign and interaction effects on advertisement judgments. Advertisements featuring Black SMM exclusively were judged as more stigmatizing than advertisements without couples. Advertisements with diverse (vs. no) couples were considered more eye-catching and motivating. There were minimal effects of couple composition and campaign on PrEP stigma, motivation, and behavior. Focus group participants corroborated concerns about the potential for PrEP advertisements to be stigmatizing, suggesting advertisements featuring Black SMM exclusively could be alienating and fuel conspiracy theories. Focus group participants generally favored diverse and less sexualized advertisements, particularly for public spaces. Findings collectively highlight the potential for targeted PrEP advertisements to stigmatize Black SMM and support diverse representation.

## Introduction

In the US, stark HIV disparities persist, with Black sexual minority men (SMM) adversely and disproportionately affected [1]. HIV pre-exposure prophylaxis (PrEP) can virtually eliminate the risk of sexually acquiring HIV if taken as prescribed [2]. Although the first PrEP medication was approved by the US Food and Drug Administration over a decade ago, awareness and access are still low in general [3–5], and inequitably so among Black SMM [6, 7]. Pervasive disparities in both HIV incidence and PrEP access suggest a need to prioritize Black SMM in PrEP social marketing initiatives, which often take the form of targeted advertising that includes visual representations of Black SMM. PrEP advertising has the potential to inadvertently perpetuate stigma [8], which has been identified as a potential barrier to PrEP uptake [9]. In the current mixed methods study, we systematically evaluated how featuring Black SMM in PrEP visual advertisements affects Black SMM’s perceptions about such advertisements. Additionally, we examined the impact of such advertisements on PrEP stigma, motivation, and behavior among Black SMM, and we explored Black SMM’s preferences related to PrEP visual advertising more generally.

### Background

Social marketing entails the application of commercial marketing principles and techniques to the promotion of socially beneficial behavior change, typically focusing on a given priority group or “target audience” [10, 11]. Many PrEP social marketing campaigns to date have included targeted visual advertisements prominently featuring Black SMM and other groups disproportionately affected by HIV in an effort to appeal to members of these groups. In social marketing campaigns promoting health prevention products and services, targeting is a commonly used strategy to reach the groups most affected by the respective health issue. For example, youth are featured in anti-vaping campaigns (e.g., *The Real Cost* campaign) and women are featured in breast cancer awareness campaigns (e.g., *Bring Your Brave* campaign).

Despite this common practice, when developing targeted PrEP social marketing campaigns to promote PrEP among Black SMM, stigma surrounding the health product (PrEP), the health issue it is intended to address (HIV), and the target audience (Black SMM) merits special consideration. Stigma refers to social devaluation based on a distinguishing characteristic that maintains power hierarchies within society [12, 13]. PrEP stigma, which involves the differentiation and devaluation of individuals because of their PrEP use, includes stereotypes of PrEP users as sexually promiscuous, irresponsible, and immoral [14]. Among Black SMM and other prospective PrEP users, these associations can foster expectations of negative judgments and disapproval by other people (anticipated PrEP stigma) and discourage PrEP interest and use [14–16]. HIV stigma encompasses (unfounded) fears of transmission and beliefs about irresponsibility and immorality [17, 18]. Like PrEP stigma, HIV stigma and fears of being mistaken as having HIV can discourage PrEP uptake [19]. Black SMM stigma includes sexual stereotypes related to promiscuity and disease risk [20]. Such stereotypes have also posed barriers to PrEP access, for example, reducing Black SMM’s comfort discussing their sexuality with healthcare providers [21]. Notably, these three forms of stigma—PrEP stigma, HIV stigma, and Black SMM stigma—are synergistic and can mutually reinforce one another [14]. Thus, prominently featuring Black SMM in visual advertisements promoting a health product that is stereotypically associated with sexual promiscuity (PrEP) to address a stigmatized sexual health issue (HIV) can make negative sexual stereotypes of Black SMM more salient, amplifying anticipated stigma and ultimately reducing Black SMM’s motivation to use PrEP.

The limited existing research examining targeted PrEP social marketing has revealed mixed reactions among targeted groups and other community members. On the one hand, prior research has illuminated several stigma-related concerns. Using qualitative interviews and focus groups, several researchers have found that SMM perceive the focus on SMM to perpetuate stigma [19, 22–24]. SMM have highlighted that exclusively focusing on gay men could deter PrEP use among SMM who do not identify as gay or are not open about their sexuality, in addition to excluding other people for whom PrEP is indicated [19, 25]. Black SMM have reported that targeted PrEP messaging is stressful and insinuates both that Black SMM are to blame for their HIV risk and that other races do not share that risk [19, 23, 24]. Likewise, evaluation of the *PrEP4Love* campaign—a PrEP promotional campaign in Chicago prominently featuring Black SMM and other people of color—found that it fueled existing racialized sexual stereotypes (e.g., prompting a community complaint linking Black SMM to risk and disease) and elicited concerns about stigmatization among some community members [8].

On the other hand, research has also suggested favorable reactions and outcomes of targeted PrEP social marketing. For example, some Black SMM have expressed a desire to see themselves in PrEP visual marketing materials, valuing the ability to identify with the people depicted [23, 25]. Additionally, analysis of survey data, *PrEP4Love* website clicks, and calls to an informational hotline has revealed that the *PrEP4Love* campaign encouraged widespread community awareness about PrEP, including among young sexual and gender minorities, and that it was positively associated with PrEP use and perceived peer approval of PrEP among young sexual and gender minorities [8, 26–28].

Importantly, knowledge of Black SMM’s perceptions about targeted PrEP social marketing and its impact is derived from studies examining attitudes about PrEP social marketing in general or outcomes pertaining to a single social marketing campaign. Although these past studies offer foundational insights, direct comparison of targeted and non-targeted PrEP social marketing is crucial for understanding the implications of the targeting itself. Additionally, conducting this comparison within and across multiple social marketing campaigns could account for campaign-specific factors.

### Study overview

In the current study, we used mixed methods to compare targeted and non-targeted PrEP visual advertisements adapted from three social marketing campaigns. Specifically, we examined Black SMM’s differential responses to advertisements that featured a Black SMM couple (targeted advertisements) and advertisements featuring a Black heterosexual couple, diverse couples, or no couples.

**Fig 1:**
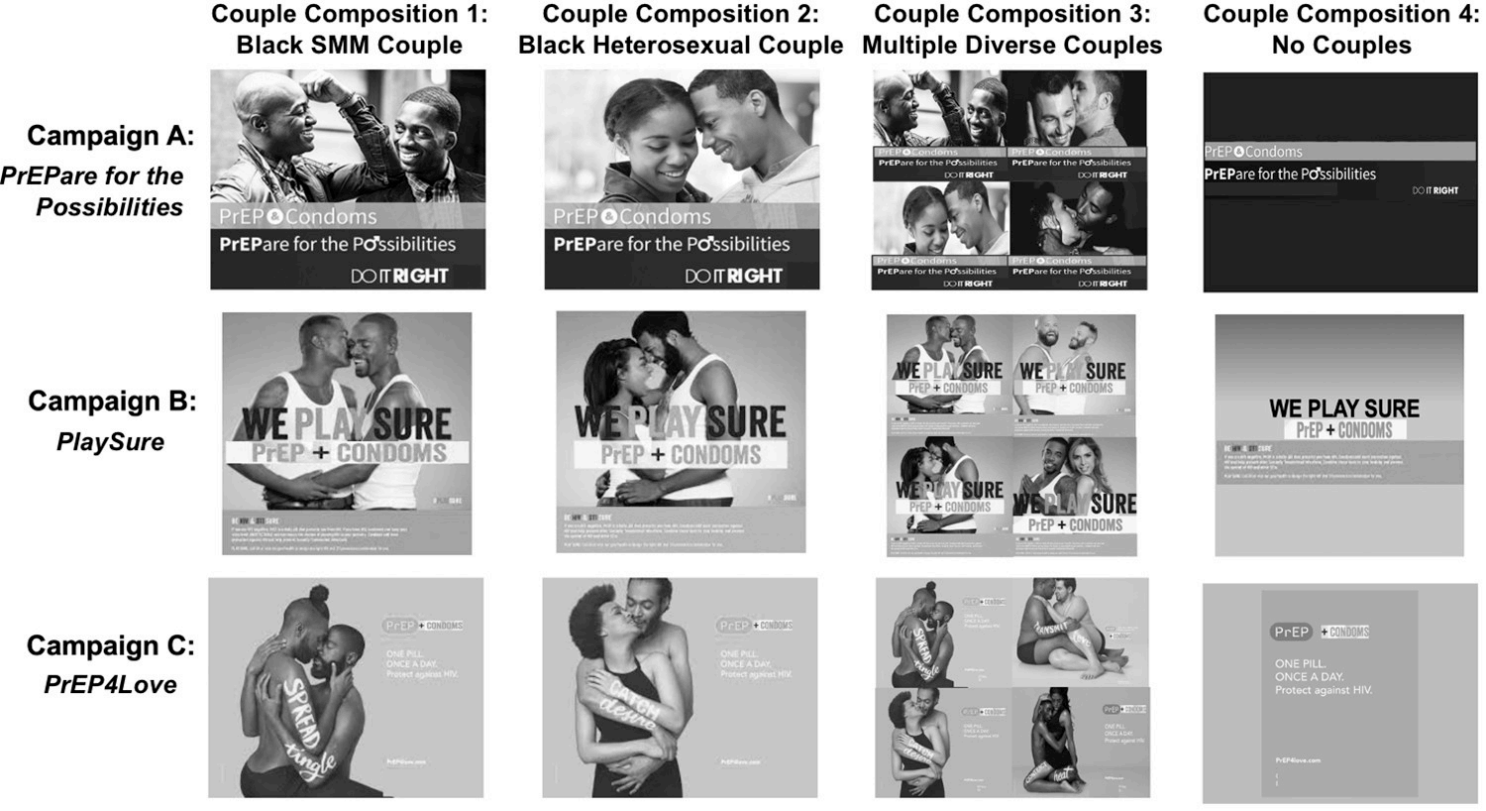
Visual stimuli used in 4x3 experimental design. Advertisements were adapted from three existing campaigns: *PrEPare for the Possibilities*, *PlaySure*, and *PrEP4Love*. *Images were printed with permission from the DC Department of Health*, *New York City Department of Health and Mental Hygiene*, *and Illinois PrEP Working Group under a CC BY license*. SMM = sexual minority men.

The campaigns included: (a) the *Prepare for the Possibilities* campaign, which depicted couples kissing or otherwise interacting affectionately above the text “PrEP & Condoms,” “PrEPare for the Possibilities,” and “Do It Right”; (b) the *PlaySure* campaign, which portrayed couples embracing or otherwise in close contact, overlayed with the text “We Play Sure” and “PrEP + Condoms”; and (c) the aforementioned *PrEP4Love* campaign, which featured couples embracing alongside an image representing PrEP and the text “ONE Pill,” “ONCE A DAY,” and “Protect against HIV.” The couples in the *PrEP4Love* campaign also displayed body art pairing words traditionally connoting infection with sex-positive outcomes (e.g., “SPREAD tingle,” “CATCH desire”). The adapted versions of the campaign advertisements that we used in the current study are displayed in **Fig 1,** configured according to the 4 (couple composition) x 3 (campaign) experimental design.

We assessed whether couple composition impacted advertisement judgments, PrEP stigma, PrEP motivation, and PrEP behavior. We tested the generalizability of the impact of couple composition by examining whether effects varied across advertisement campaign (i.e., whether campaign moderated the effects), in addition to assessing whether campaign had any independent effects. We employed a longitudinal design to enable assessment of behavioral outcomes (e.g., PrEP information seeking, PrEP initiation) occurring after exposure to the PrEP advertisements. We hypothesized that advertisements featuring a Black male couple exclusively would be perceived as more stigmatizing and otherwise rated less favorably compared with advertisements featuring other couple compositions. Our evaluation of campaign as a moderator and of PrEP stigma, motivation, and behavior as outcomes was exploratory. Therefore, we made no associated hypotheses.

In the qualitative portion of the study, we conducted four focus groups exploring Black SMM’s PrEP visual advertising preferences in general and their responses to the images displayed during the quantitative study in particular.

## Materials and methods

We used an exploratory concurrent mixed methods study design (QUAN + QUAL) that included an experiment embedded in a longitudinal online survey and four focus groups with Black SMM (2018–2019). All study procedures were reviewed and approved by the George Washington University Institutional Review Board (Protocol #031752) prior to study initiation.

### Quantitative methods (online survey experiment)

#### Survey participants

Survey participants were recruited in Washington, DC, and other East Coast US cities via dating apps, social media, email listservs, and referral by other participants. Recognizing the relevance of our recruitment materials to our experimental design, all of our recruitment materials featured a single Black man (no couples) and varied systematically by level of nudity (to approximate variable nudity depicted in the study stimuli) and by age.

Eligibility criteria for the survey included identifying as Black/African American, identifying as a “man” or “transgender man,” being 18 years of age or older, being HIV-negative or of unknown HIV status, having anal sex with a man within the past year, being able to read and answer questions in English, and—given our interest in the impact of the advertisements on initial PrEP uptake—not previously using PrEP. This information was self-reported based on initial screening during the online survey, which was administered via Qualtrics® software (Qualtrics, Provo, UT).

#### Survey procedure

**Fig 2:**
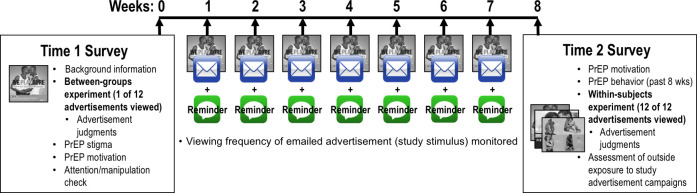
Visual overview of survey experiment. During the initial survey (Time 1), participants reported background information (e.g., sociodemographic characteristics) and were randomly assigned to view and make judgments about one of 12 visual advertisements. They also completed measures of PrEP stigma and PrEP motivation as well as an attention/manipulation check item. Eight weeks later (Time 2), after participants were re-exposed to the advertisement via weekly emails (with weekly text message reminders to view the emailed advertisement), we re-assessed PrEP motivation and evaluated PrEP behaviors occurring in the eight weeks since Time 1. At Time 2, we also presented each participant with all 12 advertisements to conduct a within-subjects comparison of advertisement judgments and assessed outside exposure to study advertisement campaigns. *Images were printed with permission from the DC Department of Health*, *New York City Department of Health and Mental Hygiene*, *and Illinois PrEP Working Group under a CC BY license*.

**Fig 2** shows a visual overview of the survey experiment. At Time 1, following initial screening and consent, participants were asked to report background information related to their sociodemographic characteristics, HIV status, and PrEP knowledge/experience.

Subsequently, participants were randomized to view one of the twelve advertisements (study stimuli) depicted in **Fig 1**. Each participant viewed a single advertisement. Advertisements systematically varied by couple composition (Black SMM couple; Black heterosexual couple; multiple diverse couples, including Black SMM as well as couples of other races and sexual orientations; or no couples) and social marketing campaign (*PrEPare for the Possibilities* campaign, *PlaySure* campaign, or *PrEP4Love* campaign) according to a 4x3 factorial design. We modified advertisements from their original form to reduce superficial differences across campaigns that could impact advertisement judgments and lead to differential evaluation of campaigns. Such modifications included concealing city names, converting all images to black-and-white coloring, incorporating “+ condoms” into the campaign in which condoms weren’t already part of the messaging (*PrEP4Love*), and removing “+ treatment” from several of the *PlaySure* advertisements. Additionally, because the original *PrEPare for the Possibilities* campaign did not include any images of a Black heterosexual couple, we superimposed an image of a Black heterosexual couple from a fourth campaign onto the advertisements for the Black heterosexual couple and multiple diverse couples conditions within the *PrEPare for the Possibilities* campaign. Participants made judgments about the advertisement that they viewed and completed a series of other measures, including PrEP stigma and motivation measures and an attention/manipulation check item.

For the next seven weeks, participants received weekly emails prompting them to view the same advertisement presented in the initial survey. They also received weekly text messages reminding them to view the emailed image. Eight weeks after the original survey, participants were emailed a link to the Time 2 survey. After completing measures of PrEP motivation and behavior, all participants were presented with the 12 advertisements sequentially, in randomized order, and made judgments about each. Participants were compensated with a $10 Amazon gift card for each of the two surveys and entered into a lottery for $500 for every week that they viewed the emailed advertisement (up to seven entries possible).

We implemented several strategies to protect our surveys from bots and other forms of fraudulent responding, such as including a CAPTCHA code and geographically restricting access based on IP address. Additionally, all incoming data were reviewed for suspicious responses or unusual response patterns and excluded as appropriate.

#### Survey measures

We collected the following *background information* at Time 1: sociodemographic characteristics (i.e., age, ethnicity, education, employment status, annual household income, and sexual orientation); HIV status; and PrEP knowledge/experience. PrEP knowledge/experience was assessed using three questions. First, participants were asked, “Before participating in this survey, had you ever HEARD OF a daily pill that an HIV-negative person can take to prevent HIV BEFORE being exposed to the HIV virus (for example, before having sex with someone who is HIV-positive)? This pill is also called HIV pre-exposure prophylaxis, PrEP, and Truvada®.” Response options were Yes, No, or I don’t know. At the time of data collection, Descovy® was not yet approved and therefore was not mentioned in the survey question. Participants were subsequently asked, “Have you ever spoken to a medical provider about starting PrEP?” with response options of Yes or No. Those who responded “Yes” were then asked, “Has a medical provider ever prescribed PrEP for you?” with response options of Yes or No. Prior PrEP use was assessed as part of the initial eligibility screening at Time 1 by asking: “Have you ever used HIV pre-exposure prophylaxis or PrEP, which is a daily pill used to prevent HIV?” with response options of “Yes, I have taken PrEP” and “No, I have never taken PrEP.” Only participants who endorsed the latter were eligible to enroll in the study and completed the survey. Initiation of PrEP use following the Time 1 survey was an outcome assessed during the Time 2 survey (described below).

Several Time 1 background variables were recoded based on response distribution to enable their inclusion in inferential analyses. Education was dichotomized as having at least a bachelor’s degree vs. less than a bachelor’s degree, employment was dichotomized as being employed full- or part-time vs. not, annual household income was dichotomized as earning $30,000 or less vs. over $30,000, and sexual orientation was dichotomized as gay vs. other. In addition, PrEP knowledge/experience was trichotomized as never heard of PrEP vs. heard of PrEP but never discussed it with a provider vs. heard of PrEP and discussed it with a provider, and this variable was dummy coded.

*Judgments of the PrEP advertisements* presented were assessed using a between-groups approach at Time 1 and a within-subjects approach at Time 2. At Time 1, participants were presented with a single, randomly assigned advertisement, which they were told was being used to advertise PrEP. They were prompted to make 16 judgments about the image using the same item stem, “Do you find the advertisement above to be…,” followed by 16 descriptive words. The 16 words were displayed in a randomized order. Participants indicated the extent to which they judged the image to be eye-catching, positive, motivating, negative, offensive, appealing, stigmatizing, helpful, acceptable, boring, informative, upsetting, relatable, memorable, pleasant, and unpleasant. An initial list of words (e.g., “positive,” “unpleasant”) was derived from prior research evaluating advertising stimuli [29] and was expanded to include words of specific interest for the present study (e.g., “stigmatizing”, “relatable”). After each word, participants made a judgment using a scale ranging from [1] Not at all to [5] Extremely.

At Time 2, participants were presented with all 12 images and prompted to make seven judgments about each of the images using the same scale ranging from [1] Not at all to [5] Extremely. The 12 advertisements were displayed and rated sequentially in a randomized order. Participants indicated the extent to which each image was eye-catching, positive, motivating, negative, offensive, appealing, and stigmatizing. We asked participants to report fewer judgments at Time 2 compared with Time 1 to reduce participant response burden and fatigue given that we were asking each participant to rate 12 different images rather than a single image.

*PrEP stigma* was assessed using the PrEP Anticipated Stigma Scale, a measure that contains two subscales: Negative PrEP Stereotypes and PrEP Disapproval by Others [30]. For both subscales, participants responded to items by rating their agreement from [1] Strongly disagree to [4] Strongly agree. The five-item Negative PrEP Stereotypes subscale (Cronbach’s α = .78) included items such as: “People would assume that I slept around if they knew I took PrEP” and “People would assume that I am gay if they knew that I took PrEP.” The three-item PrEP Disapproval by Others subscale (Cronbach’s α = .64) included items such as: “My sexual partner(s) would approve of me taking PrEP” and “My family would approve of me taking PrEP.” Items were reverse-scored as needed. Mean subscale scores were then calculated, with scores potentially ranging from 1 to 4. Higher values indicated greater stigma (i.e., stronger endorsement of negative PrEP stereotypes and lower perceived approval of others, respectively).

*PrEP motivation* was assessed using three items, including: PrEP interest (“How interested are you in learning more about PrEP?” with a response scale ranging from [1] Not at all interested to [5] Extremely interested), PrEP willingness (“How likely would you be to take PrEP if it were available for free?” with a response scale ranging from [1] Definitely would not take PrEP to [5] Definitely would take PrEP), and perceived PrEP candidacy (“Do you believe that you are a good candidate for PrEP?” with a response scale ranging from [1] No, I am definitely NOT a good candidate to [4] Yes, I am definitely a good candidate). The three motivation items were asked at both Time 1 and Time 2. Participants who reported that they had initiated PrEP between Time 1 and Time 2 were not directly asked about PrEP willingness at Time 2, but 10 of 11 were coded as [5] (Definitely would take PrEP) for the item based on their decision to initiate PrEP and report that they were currently taking PrEP. The single participant who reported that he initiated PrEP but was not currently taking it (reason specified: “I never took prep”) was omitted from analyses related to PrEP motivation at Time 2.

*PrEP behavior* was assessed with four items, including: PrEP contemplation (“Over the past 8 weeks, how often did you think about PrEP?” with a response scale ranging from [1] Never to [4] Often), PrEP information-seeking (“Over the past 8 weeks, how often did you try to find out more information about PrEP?” with a response scale ranging from [1] Never to [4] Often), PrEP discussion (“Over the past 8 weeks, how often did you talk about PrEP?” with a response scale ranging from [1] Never to [4] Often) and PrEP initiation (“Over the past 8 weeks, did you start taking PrEP?” with response options Yes or No). Those participants who reported initiating PrEP were asked whether they were currently using PrEP and, if not, to specify their reason(s) for discontinuing it.

The *attention/manipulation check* item, which was administered toward the end of the Time 1 survey, stated: “Earlier in the survey, we showed you an image of a PrEP advertisement and asked you to rate how eye-catching it was, how offensive it was, and so on. Who was in the ad image you saw?,” to which participants could indicate: only a man and a woman, only two men, multiple couples, or not applicable (no people in the image). Participants were considered to have passed the attention/manipulation check if they answered this question correctly based on the couple composition in the advertisement that they were randomly assigned to view.

At Time 2, after advertisement judgments had been completed, participants reported *outside exposure to study advertisement campaigns*. Specifically, we asked whether they had been exposed to each of the three advertisement campaigns (*PrEPare for the Possibilities*, *PlaySure*, and *PrEP4Love*) ever in their lifetime (before or during the study) outside of the surveys, text messages, and emails that were part of the study. Those who reported outside exposure to one or more of the campaigns were asked to specify the timing (before or after starting the study).

Between Time 1 and Time 2, we monitored *advertisement (study stimulus) viewing frequency*. Specifically, we counted the views of images sent in weekly emails using third-party software (ActiveCampaign™). Based on the frequency distribution, we trichotomized number of views as 1–2, 3–10, or 11+ and dummy coded the variable for inclusion in inferential analyses involving Time 2 outcomes.

#### Survey data analysis

We calculated frequencies, means, and standard deviations to describe the sample and measures of interest. We conducted Pearson correlations to examine bivariate associations among the 16 advertisement judgments assessed at Time 1 and among the PrEP stigma, motivation, and behavior outcomes assessed at Times 1 and 2.

For the between-groups comparisons, we conducted two-way MANCOVAs examining couple composition, campaign, and interaction effects of Time 1 advertisement viewing relative to five sets of outcomes: Time 1 advertisement judgments; Time 1 PrEP stigma (Negative PrEP Stereotypes and PrEP Disapproval by Others mean subscale scores); Time 1 PrEP motivation (PrEP interest, PrEP willingness, and perceived PrEP candidacy); Time 2 PrEP motivation (PrEP interest, PrEP willingness, and perceived PrEP candidacy); and Time 2 PrEP behavior (PrEP contemplation, PrEP information-seeking, and PrEP discussion). We used Pillai’s Trace (*V*) to determine the significance of the effect of couple composition, campaign, and their interaction on each set of outcomes (*p* < .05). Where multivariate effects were significant, we performed *post hoc* pairwise comparisons applying the Sidak adjustment for multiple comparisons. We intended to analyze the remaining PrEP behavior (PrEP initiation), a dichotomous variable, separately from the other behaviors using logistic regression. However, because of the small number of participants who initiated PrEP during the eight-week follow-up period (*n* = 11), 50% of cells had expected counts less than five. Therefore, we were unable to conduct a logistic regression (which would have allowed inclusion of covariates) or a chi-square test to statistically evaluate differences in PrEP initiation by couple condition or campaign.

Background variables that were adjusted for in multivariate models for the between-groups comparisons were those that were conceptually and empirically associated with advertisement judgments, PrEP stigma, PrEP motivation, and/or PrEP behavior: age, ethnicity, education, employment, sexual orientation, and PrEP knowledge/experience. To control for the potential effect of differential levels of study stimulus exposure, we further adjusted for advertisement (study stimulus) viewing frequency for analyses involving Time 2 outcomes (PrEP motivation and behavior).

When examining Time 1 advertisement effects (MANCOVAs), we initially restricted the analytic sample to participants who passed the attention/manipulation check. This was done to ensure that all participants had adequately attended to the content of the advertisement to which they were randomly assigned. We subsequently repeated these analyses with the full, unrestricted sample to determine whether the same pattern of results emerged.

For the within-subjects comparisons, we conducted a two-way repeated-measures MANCOVA examining couple composition, campaign, and interaction effects of Time 2 advertisement viewing relative to the set of seven advertisement judgments. We used Pillai’s Trace (*V*) to determine significance (*p* < .05). Where multivariate effects were significant based on the true doubly-multivariate model, we performed *post hoc* pairwise comparisons applying the Sidak adjustment for multiple comparisons. Background variables that were adjusted for in multivariate models were those that were conceptually associated with Time 2 advertisement judgments and empirically associated when included as a single covariate in the repeated measures model: age, ethnicity, PrEP knowledge/experience, and advertisement (study stimulus) viewing frequency during the eight-week follow-up period. Couple composition and campaign of the advertisements viewed at Time 1 were not significantly associated with judgments of the advertisements viewed at Time 2 and therefore were not included in the repeated measures MANCOVA models.

### Qualitative methods (focus groups)

#### Focus group participants

Focus group participants were recruited using the same methods as survey recruitment (dating apps, social media, email listservs, and referral by other participants); however, because the focus groups were held in person, recruitment efforts were primarily focused in the Washington, DC/Baltimore metro area. Individuals who participated in the survey portion of the study were invited to participate in the focus groups as well. Focus group eligibility criteria were similar to survey eligibility criteria except that focus group eligibility was restricted to cisgender men and was not restricted by PrEP status. Whereas only PrEP-inexperienced participants were included in the survey because we were studying motivational and behavioral outcomes of advertisement viewing with an emphasis on initial PrEP uptake, individuals with no prior PrEP use and those with PrEP experience were eligible for focus groups to elicit multiple perspectives. Focus group eligibility screening was conducted online via Qualtrics® software and by phone.

#### Focus group procedure

Focus groups were scheduled based on the contact information and preferences expressed by eligible individuals during the screening process. All focus groups were led by two facilitators (SR and DXM) and held in a private room in a university building accessible via public transportation. At the outset of each focus group, the facilitators informed attendees of the risks and rights associated with study participation and obtained verbal consent from all participants. Subsequently, they asked participants to complete a brief background questionnaire, which included questions related to participants’ sociodemographic characteristics, HIV status, and PrEP knowledge/experience.

Following questionnaire completion, facilitators led the semi-structured discussion using a thematically organized guide that contained lead questions and follow-up prompts. Before asking any questions, they provided a brief description of PrEP to ensure that all participants had a basic understanding of the regimen, side effects, its approval by the US Food and Drug Administration, and its demonstrated success with multiple populations, including SMM, heterosexual men and women, and people who inject drugs. Thereafter, participants were asked to describe their preferences for PrEP visual advertisements, including the imagery that they would include and their perspective on targeting the advertisements to men who have sex with men. After soliciting these general preferences, participants were presented with a poster displaying a 4x3 grid of visual advertisements like the one depicted in **Fig 1**. Rows and columns of the grid were labeled simply with letters and numbers (Row A-C, Column 1–4) rather than campaigns and couple compositions. We deliberately varied the ordering of rows and columns across the four focus groups. Participants were prompted to share their perspective on the acceptability, comprehensibility, and relatability of the advertisements displayed in the poster and their perception of the advertisements’ impact on PrEP motivation and behavior. They were encouraged to draw direct comparisons across advertisements and explain differential preferences. At the conclusion of each focus group, participants were compensated with $50 and provided with a resource packet, which included a directory of local PrEP providers. All focus groups were audio-recorded and lasted 66–78 minutes.

#### Focus group data analysis

Thematic analysis of focus group data was guided by the Framework Method, which includes transcription, data familiarization, coding, development of a working analytic framework, framework application, data charting, and interpretation [31]. All audio recordings of focus group discussions were transcribed verbatim, and transcripts were uploaded into NVivo 11 for analysis. Two coders (DAK and SR), one of whom co-facilitated all focus groups (SR), drafted an initial analytic framework. They subsequently refined the framework in consultation with the principal investigator (SKC) using an iterative process, which entailed the coders independently coding transcripts according to the drafted analytic framework and then reconvening to discuss the coding and revise the analytic framework, adding, consolidating, and clarifying codes as needed. The coders documented all coding conventions and decisions. Once the framework was finalized, the two coders independently coded two of the four transcripts using the finalized framework and then compared their coding to establish inter-rater reliability [32, 33]. After reliability had been established, the remaining transcripts were independently coded by a single coder.

For the analysis presented here, the principal investigator reread all transcripts and charted the data. She used the chart to guide data interpretation and select illustrative quotes. Quotes are presented along with the corresponding participant and focus group numbers. Additionally, each quote is designated as occurring before or after the 4x3 grid of visual advertisements was presented to participants.

Of note, this article presents participants’ perspectives on the visual elements of the advertisements, including targeted images. A different article [34] presents participants’ responses to the textual elements of the advertisements, which are not included here.

#### Reflexivity

Throughout the study, research team members were mindful of how their personal backgrounds, experiences, and perspectives could influence the work, including the qualitative themes identified and interpretations drawn from the data [35]. The research team was diverse with respect to race/ethnicity, sexual orientation, and gender. The team was led by a White, heterosexual, cisgender woman, who guided all aspects of the study and led the charting and interpretation of qualitative data. Focus groups were led by a White, genderqueer/genderfluid male who is attracted to other males and a South Asian, queer, cisgender woman. The latter contributed to the drafting of the initial qualitative analytic framework, as did a White, gay, cisgender man. Team members included researchers from multiple academic disciplines and the local health department. Multiple team members had previously investigated sexual stereotypes and stigma encountered by Black SMM. All had prior experience conducting HIV prevention research with Black SMM and supported the goal of optimizing PrEP social marketing to increase PrEP awareness and access for Black SMM.

## Results

### Survey

#### Survey sample overview

The Time 1 analytic sample included 96 participants who were randomly assigned to view one of the 12 advertisements and completed the initial survey. Participant background characteristics are presented in **Table 1**. All participants at Time 1 identified as a “man,” and none identified as a “transgender man.” They ranged in age from 19–72 years (*M*[*SD*] = 32[10.7]). Most were employed full- or part-time (83.3%), identified as gay (78.1%) or bisexual (14.6%), and had previously heard of PrEP (67.7%). Although none of the participants had previously taken PrEP (in accordance with study eligibility criteria), 25.0% reported previously discussing PrEP with a healthcare provider and 4.2% reported previously obtaining a prescription for it from a provider.

**Table 1:** Sample characteristics.

	Time 1 Survey^a^	Time 2 Survey^a^	Focus Groups^b,c^
	*n* (%)	*n* (%)	*n* (%)
**Age**			
18–25 years	26 (27.1)	20 (27.4)	3 (16.7)
26–45 years	57 (59.4)	45 (61.6)	13 (72.2)
46+ years	13 (13.5)	8 (11.0)	2 (11.1)
**Ethnicity**			
Non-Latinx/Hispanic	60 (62.5)	38 (52.1)	16 (88.9)
Latinx/Hispanic	36 (37.5)	35 (47.9)	2 (11.1)
**Education** ^**d**^			
<Bachelor’s degree	60 (62.5)	49 (67.1)	-
≥Bachelor’s degree	36 (37.5)	24 (32.9)	-
**Employment Status** ^**d**^			
Employed (full-time or part-time)	80 (83.3)	62 (84.9)	-
Unemployed	9 (9.4)	6 (8.2)	-
Other	7 (7.3)	5 (6.8)	-
**Annual Household Income** ^**d**^			
<$10,000	9 (9.4)	6 (8.2)	-
$10,000-$29,999	11 (11.5)	5 (6.8)	-
$30,000-$49,999	24 (25.0)	21 (28.8)	-
$50,000-$69,999	33 (34.4)	29 (39.7)	-
$70,000-$89,999	10 (10.4)	5 (6.8)	-
≥$90,000	9 (9.4)	7 (9.6)	-
**Sexual Orientation**			
Gay	75 (78.1)	60 (82.2)	11 (61.1)
Bisexual	14 (14.6)	8 (11.0)	7 (38.9)
Heterosexual	1 (1.0)	0 (0.0)	0 (0.0)
Other	6 (6.3)	5 (6.8)	0 (0.0)
**HIV Status**			
Negative	85 (88.5)	63 (86.3)	18 (100.0)
Unknown	11 (11.5)	10 (13.7)	0 (0.0)
**PrEP Knowledge/Experience Prior to Study** ^**e,f**^			
Heard of PrEP	65 (67.7)	44 (60.3)	15 (88.2)
Discussed PrEP with provider^d^	24 (25.0)	16 (21.9)	-
Obtained PrEP prescription^d^	4 (4.2)	3 (4.1)	-
Used PrEP^f^	0 (0.0)	0 (0.0)	8 (47.1)
**PrEP Initiation Between Time 1 and Time 2 Surveys** ^**d,g**^			
Initiated and currently using PrEP	-	10 (13.9)	-
Initiated and discontinued PrEP	-	1 (1.4)	-
**Outside Exposure to Study Advertisement Campaigns** ^**d,e,h**^			
None of the three campaigns	58 (80.6)	42 (57.5)	-
*PrEPare for the Possibilities*	-	17 (23.3)	-
*We Play Sure*	-	16 (21.9)	-
*PrEP4Love*	-	8 (11.0)	-
**Total**	**96 (100.0)**	**73 (100.0)**	**18 (100.0)**

^a^Characteristics were reported during the Time 1 survey unless otherwise indicated.

^b^Characteristics were reported at the time of the focus group.

^c^Nine (50.0%) focus group participants reported also participating in the survey phase of the study. Three (16.7%) reported not knowing whether they had done so.

^d^Information was not collected on the focus group background questionnaire.

^e^Categories were not mutually exclusive.

^f^Focus group percentages were calculated with a denominator of *n* = 17 due to missing data.

^g^PrEP initiation between the Time 1 and Time 2 surveys was assessed during the Time 2 survey. Percentages were calculated with a denominator of *n* = 72 due to missing data.

^h^Outside exposure to study advertisement campaigns was assessed during the Time 2 survey. Time 1 survey values reflect participants who reported that outside exposure occurred before starting the study. Time 1 percentages were calculated with a denominator of *n* = 72 because data were reported during the Time 2 survey (n = 73) and the participant who didn’t know/remember the timing of outside advertisement exposure was coded as missing.

Of the 96 participants at Time 1, the Time 2 analytic sample included the 73 participants (76.0%) who completed the follow-up survey eight weeks later. Participants who completed the Time 2 survey did not significantly differ from participants who did not complete the Time 2 survey by assigned advertisement (couple composition or campaign) or most background characteristics. However, a higher percentage of those who completed the Time 2 survey versus those who did not were Latinx (*X*^2^ [1, N = 96] = 14.18, *p* < .001), earned over $30,000/year (*X*^2^ [1, N = 96] = 6.14, *p* = .013), and had not previously heard of PrEP (*X*^2^ [1, N = 96] = 7.70, *p* = .006). When asked at Time 2, 19.4% reported having been exposed to at least one of the three advertisement campaigns outside of study participation before starting the study at Time 1 and 42.5% reported having been exposed outside of study participation before or after starting the study.

Of the 96 participants in the full analytic sample at Time 1, 90 (93.8%) passed the attention/manipulation check item required to be included in analyses examining experimental effects of Time 1 advertisement exposure (couple composition and campaign) on advertisement judgments, PrEP stigma, and PrEP motivation at Time 1. Of the 73 participants in the full analytic sample at Time 2, 69 (94.5%) passed the attention/manipulation check item required to be included in analyses examining experimental effects of Time 1 advertisement exposure on PrEP motivation and behavior at Time 2.

Of the 73 participants in the full analytic sample at Time 2, the number of times the emailed advertisement was viewed over the eight-week follow up period ranged from 1 to 36; 38.4% viewed the advertisement 1–2 times, 28.8% viewed it 3–10 times, and 32.9% viewed it 11 or more times.

### Effects of couple composition and campaign on advertisement judgments: Between-groups analysis (Time 1 advertisement viewing)

**Fig 3:**
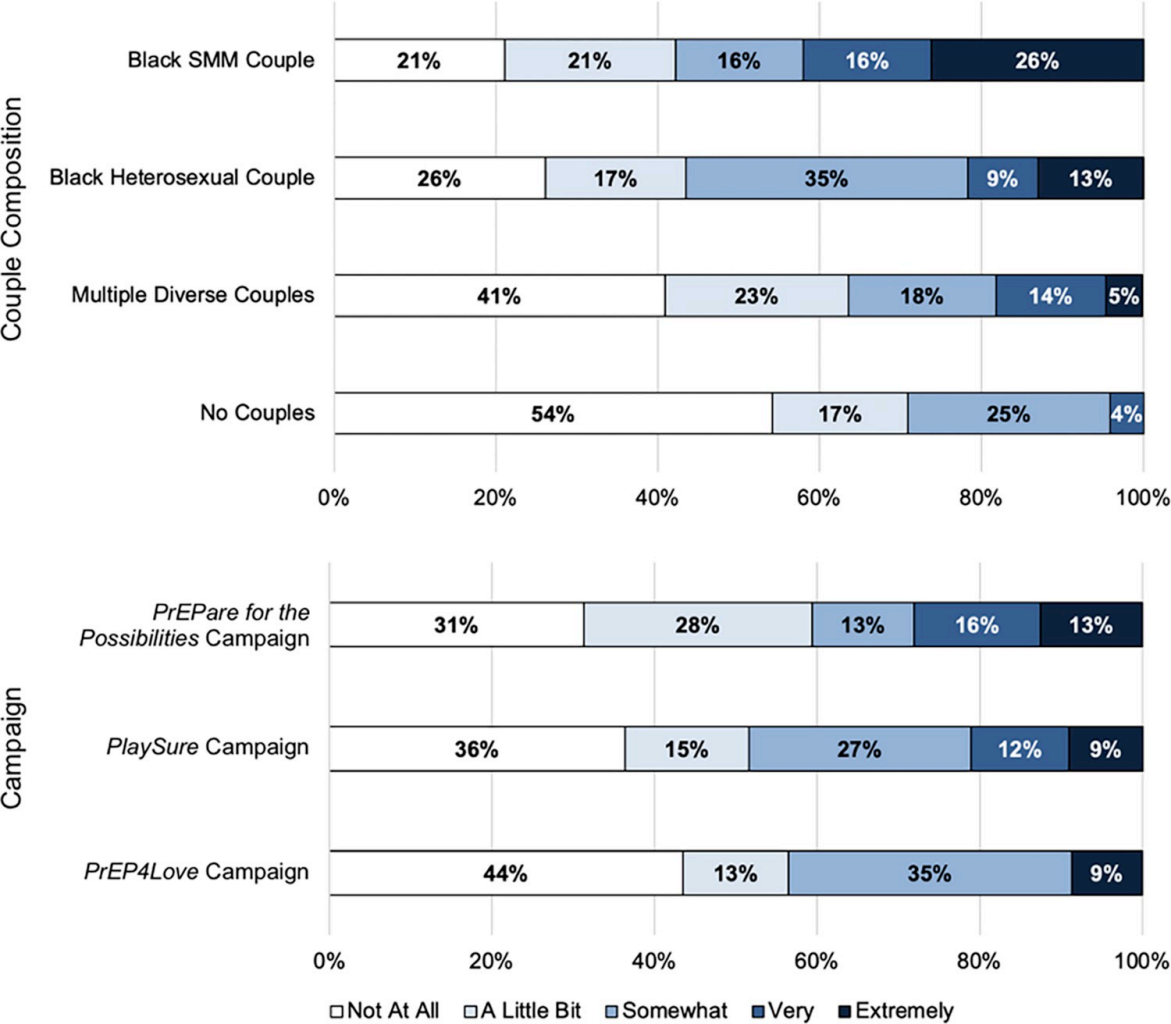
Participant judgment of advertisements as stigmatizing (Time 1). At Time 1, participants were randomly assigned to view one of 12 advertisements and to make 16 judgments about it, including judging the extent to which they perceived it to be stigmatizing. Participants rated their judgment of the advertisement as stigmatizing on a scale ranging from [1] Not at all to [5] Extremely. Frequency distributions of participant ratings are displayed by couple composition, collapsing across campaigns, and by campaign, collapsing across couple compositions. SMM = sexual minority men.

Participant ratings of the advertisements as stigmatizing are summarized in **Fig 3**. Notably, almost half (42.1%) of the participants who viewed an advertisement featuring Black SMM exclusively rated the advertisement as “very” or “extremely” stigmatizing. Means, standard deviations, and bivariate correlations of all advertisement judgments at Time 1 are presented in **S1 Table**.

**Fig 4:**
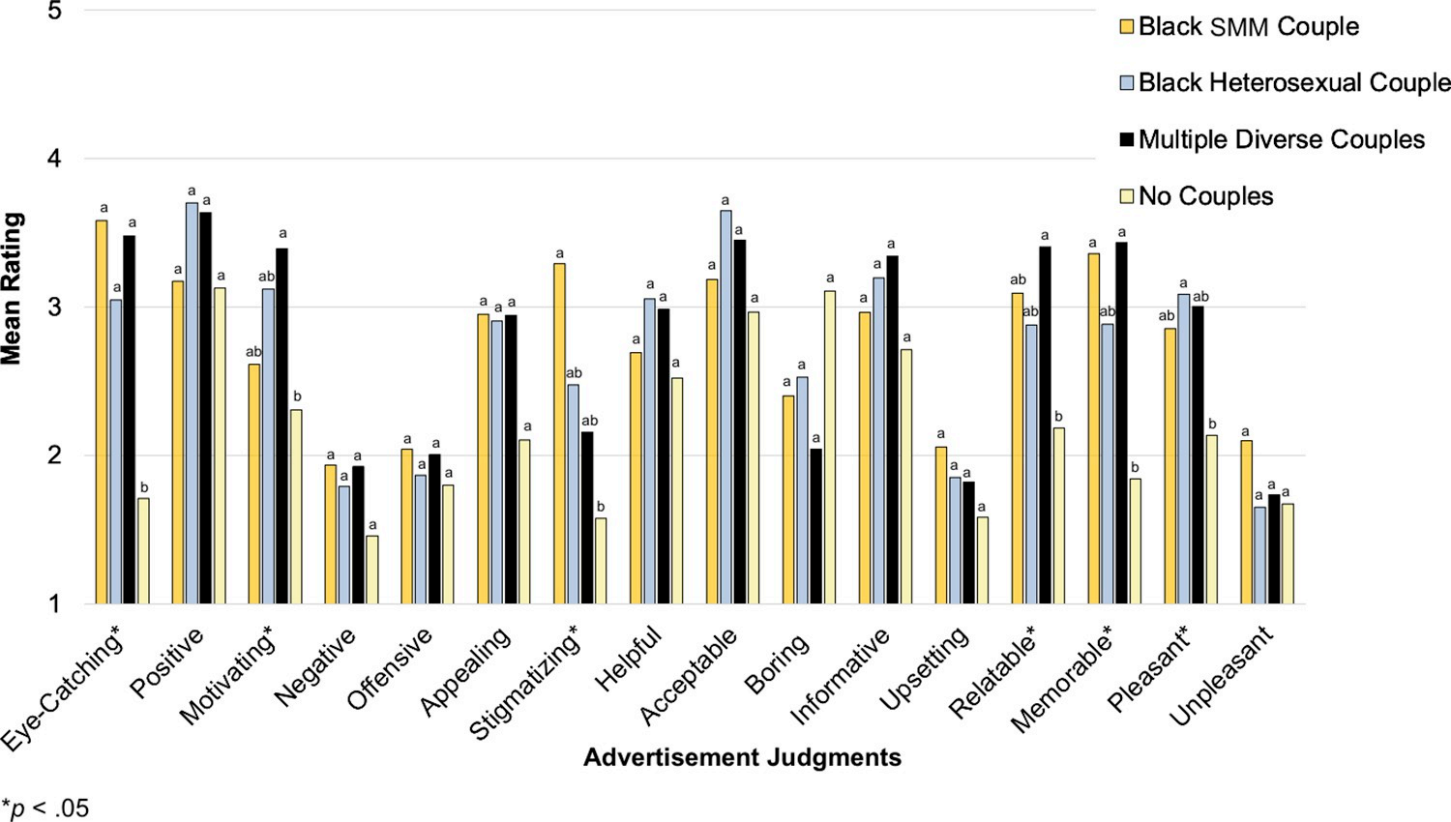
Advertisement judgments by couple composition (Time 1 between-groups comparisons). At Time 1, each participant viewed one advertisement and made 16 judgments about it on a scale ranging from [1] Not at all to [5] Extremely. Adjusted mean ratings are displayed by couple composition, collapsing across campaigns. Means were adjusted for age, ethnicity, education, employment, sexual orientation, and PrEP knowledge/experience. Significant differences were identified with respect to six of 16 judgments. For each judgment, different letters (and no shared letters) indicate statistically significant mean differences between two couple compositions. For example, as indicated by differing letters (a and b), advertisements featuring diverse couples were rated as significantly more motivating than advertisements featuring no couples. As indicated by shared letters (ab), mean ratings of Black SMM and Black heterosexual couples did not significantly differ from each other, diverse couples, or no couples with respect to this judgment. SMM = sexual minority men.

**Table 2** displays results of the MANCOVAs examining the partial, conditional, and interaction effects of couple composition and campaign on advertisement judgments. There was a significant couple composition effect but no significant campaign or interaction effects. **Fig 4** presents adjusted mean ratings across 16 judgments for the four couple compositions, collapsing across campaigns. *Post hoc* pairwise comparisons applying the Sidak adjustment for multiple comparisons revealed significant differences by couple composition across six of 16 judgments: eye-catching, motivating, stigmatizing, relatable, memorable, and pleasant. Advertisements featuring Black SMM exclusively were perceived as the most stigmatizing of the couple compositions presented and significantly more stigmatizing than advertisements featuring no couples. Apart from this judgment, a general pattern emerged with advertisements featuring no couples being rated the least favorably. Advertisements featuring diverse couples were rated as the most motivating, relatable, and memorable—significantly more so than advertisements with no couples.

**Table 2:** Effects of couple composition and campaign on advertisement judgments and PrEP stigma, motivation, and behavior (MANCOVAs).

Time 1: Immediately After First Viewing Advertisement																								
	Advertisement Judgments								PrEP Stigma								PrEP Motivation							
	Model 1^a^				Model 2^a^				Model 1^a^				Model 2^a^				Model 1^a^				Model 2^a^			
	*V*	*F*	*DF*	*p*	*V*	*F*	*DF*	*p*	*V*	*F*	*DF*	*p*	*V*	*F*	*DF*	*p*	*V*	*F*	*DF*	*p*	*V*	*F*	*DF*	*p*
Couple Composition	.97	1.63	48, 165	.013	1.00	1.54	48, 147	.027	.03	.41	6, 150	.869	.04	0.45	6, 138	.846	.02	.15	9, 231	.998	.02	.19	9, 213	.996
Campaign	.58	1.39	32, 108	.107	.64	1.39	32, 96	.110	.04	.71	4, 150	.583	.04	0.78	4, 138	.541	.10	1.36	6, 152	.236	.08	.98	6, 140	.439
Couple Composition x Campaign	-	-	-	-	1.47	1.05	96, 312	.367	-	-	-	-	.18	1.16	12, 138	.316	-	-	-	-	.19	.80	18, 213	.705
Time 2: Eight Weeks After First Viewing Advertisement																								
	PrEP Motivation								PrEP Behavior															
	Model 1^a,b^				Model 2^a,b^				Model 1^a,b^				Model 2^a,b^											
	*V*	*F*	*DF*	*p*	*V*	*F*	*DF*	*p*	*V*	*F*	*DF*	*p*	*V*	*F*	*DF*	*p*								
Couple Composition	.18	1.13	9, 156	.345	.21	1.17	9, 138	.320	.13	.81	9, 156	.607	.17	.91	9, 138	.515								
Campaign	.20	1.92	6, 102	.084	.26	2.24	6, 90	.046	.26	2.52	6, 102	.026	.23	1.98	6, 90	.077								
Couple Composition x Campaign	-	-	-	-	.34	.99	18, 138	.476	-	-	-	-	.36	1.04	18, 138	.417								

*Note*. For each set of outcomes, Model 1 represents partial effects and Model 2 represents conditional and interaction effects.

^a^All models were adjusted for age, ethnicity, education, employment, sexual orientation, and PrEP knowledge/experience.

^b^Time 2 models were also adjusted for advertisement (study stimulus) viewing frequency during the 8-week follow-up period.

### Effects of couple composition and campaign on advertisement judgments: Within-subjects analysis (Time 2 advertisement viewing)

**Fig 5:**
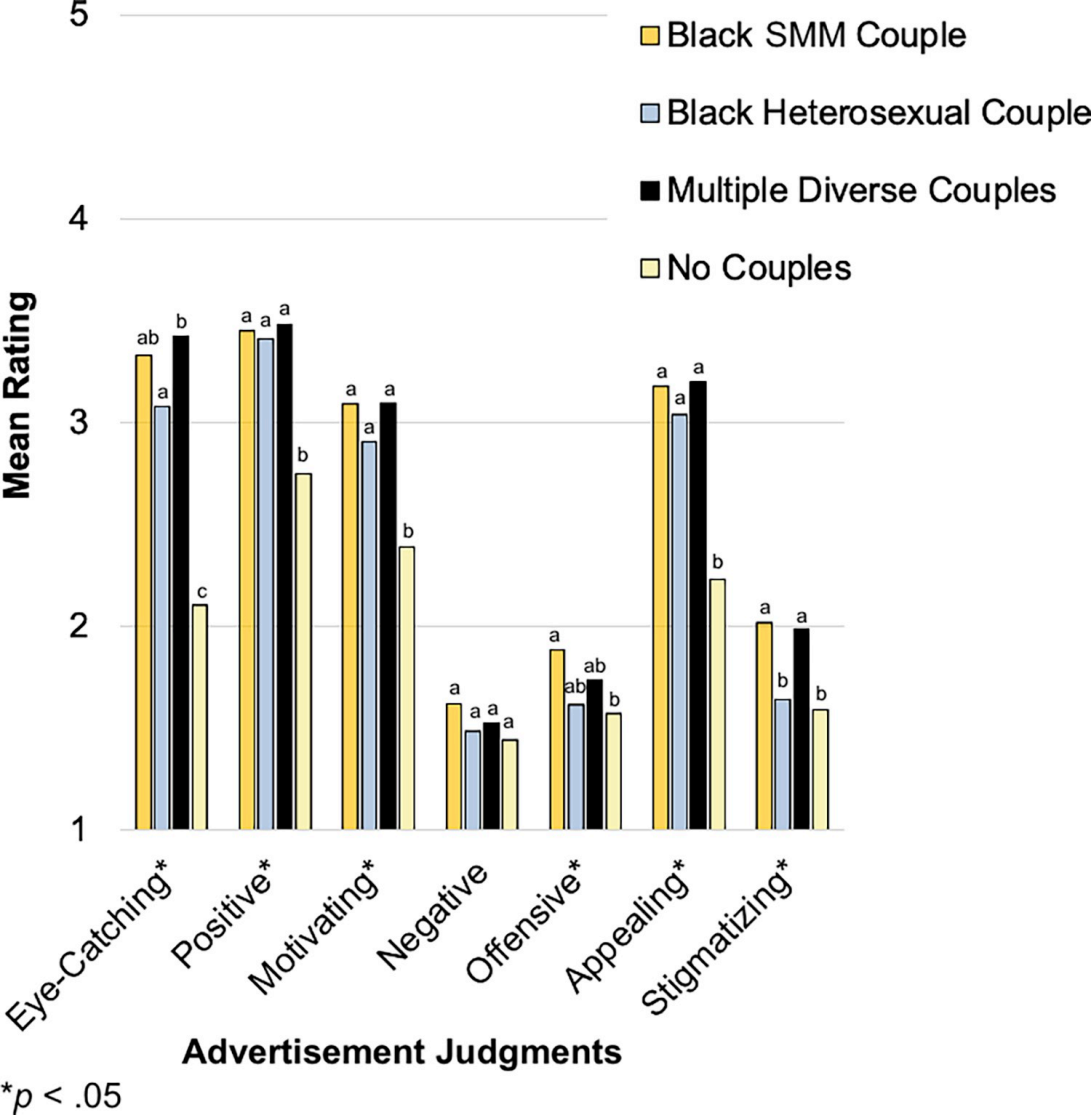
Advertisement judgments by couple composition (Time 2 within-subjects comparisons). At Time 2, each participant viewed all 12 visual advertisements and made seven judgments about each on a scale ranging from [1] Not at all to [5] Extremely. Adjusted mean ratings are displayed by couple composition, collapsing across campaigns. Means were adjusted for age, ethnicity, PrEP knowledge/experience, and advertisement (study stimulus) viewing frequency during the eight-week follow-up period. Significant differences were identified with respect to six of seven judgments. For each judgment, different letters (and no shared letters) indicate statistically significant mean differences between two couple compositions. SMM = sexual minority men.

The two-way repeated measures MANCOVA examining the effects of couple composition, campaign, and their interaction on advertisement judgments revealed a couple composition effect (*V* = .63 *F*[21, 25] = 2.01, *p* = .048) and interaction effect (*V* = .99, *F*[42, 4] = 7.09, *p* = .034) but no significant campaign effect (*V* = .45, *F*[14, 32] = 1.85, *p* = .074). **Fig 5** presents adjusted mean ratings across seven judgments for the four couple compositions, collapsing across campaigns. *Post hoc* pairwise comparisons applying the Sidak adjustment for multiple comparisons revealed significant differences across six of seven judgments: eye-catching, positive, motivating, offensive, appealing, and stigmatizing. Advertisements featuring Black SMM exclusively and those featuring diverse couples were perceived as the most stigmatizing and significantly more stigmatizing than advertisements featuring a heterosexual couple or no couples; however, the means were generally low on these dimensions, and the two types of advertisements (those featuring Black SMM exclusively and those featuring diverse couples) were rated as among the most eye-catching, positive, motivating, and appealing. Likewise, although advertisements featuring no couples were perceived as significantly less stigmatizing as those featuring Black SMM exclusively or diverse couples, they were also rated as the least eye-catching, positive, motivating, and appealing of the four couple compositions.

**Fig 6:**
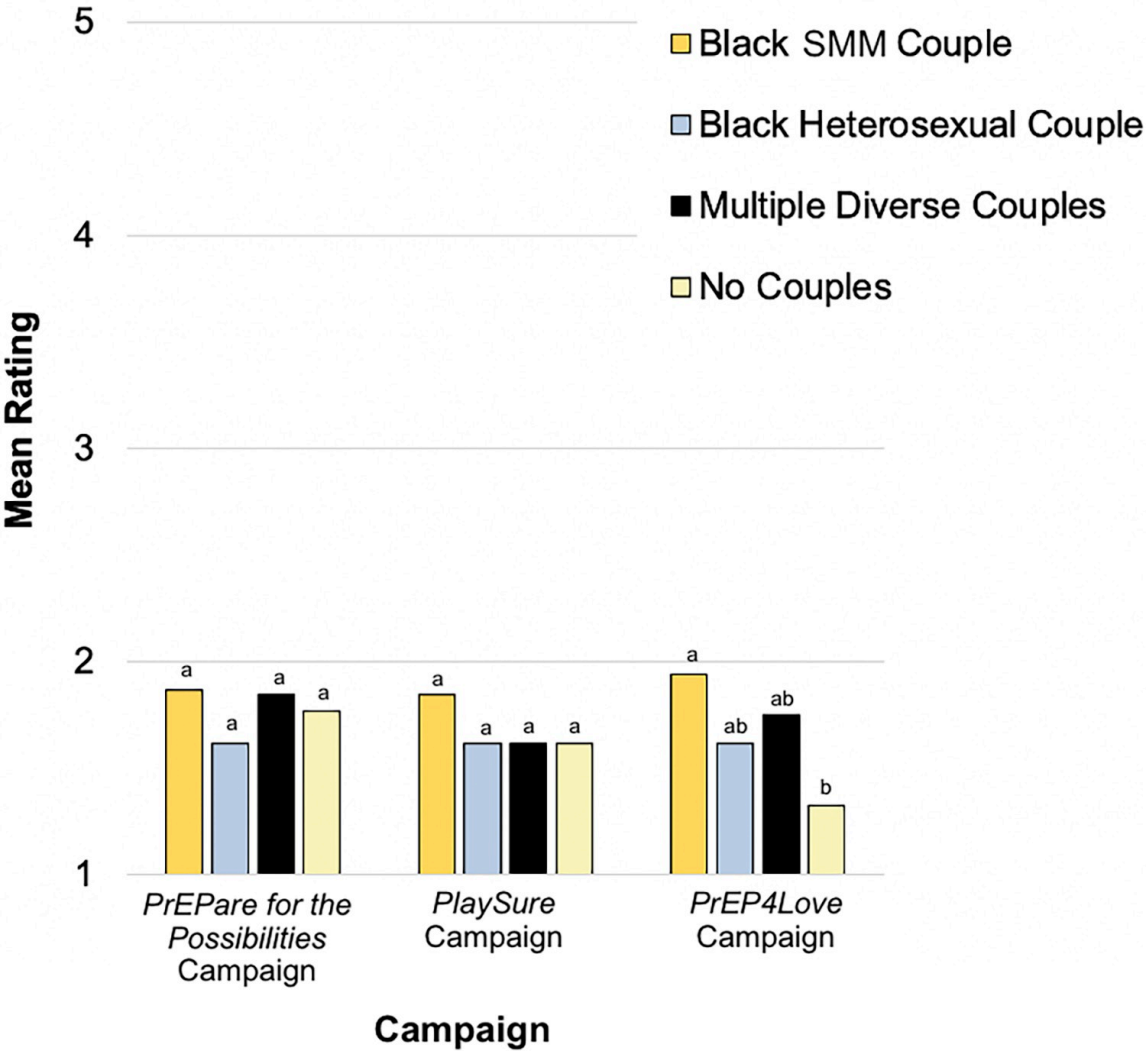
Participant judgment of advertisements as offensive by couple composition and campaign (Time 2 within-subjects comparisons). At Time 2, each participant viewed all 12 visual advertisements and made seven judgments about each, including judging the extent to which they perceived it to be offensive. Participants rated their judgment of each advertisement as offensive on a scale ranging from [1] Not at all to [5] Extremely. Adjusted mean ratings are displayed by couple composition and campaign. Means were adjusted for age, ethnicity, PrEP knowledge/experience, and advertisement (study stimulus) viewing frequency during the eight-week follow-up period. Within each campaign, different letters (and no shared letters) indicate statistically significant mean differences between two couple compositions. SMM = sexual minority men.

**Fig 6** displays adjusted mean ratings by both couple composition and campaign for the single advertisement judgment for which a significant interaction was detected: offensive (*F*[4.10, 184.67] = 2.48, p = .044, applying Greenhouse-Geisser correction for violation of sphericity). Contrasts indicated that, particularly in the *PrEP4Love* campaign, the advertisement featuring Black SMM exclusively was rated as more offensive than the advertisement with no couples. However, the means of both were relatively low (1.94 and 1.33 out of 5, respectively).

### Effects of couple composition and campaign on PrEP stigma, motivation, and behavior

Means, standard deviations, and bivariate correlations of PrEP stigma, motivation, and behavior outcomes are presented in **S2 Table**. **Table 2** displays results of the MANCOVAs examining the partial, conditional, and interaction effects of couple composition and campaign on these outcomes. There were no significant effects on PrEP stigma or motivation at Time 1. At Time 2, there were no significant couple or interaction effects on PrEP motivation or behavior. There were, however, significant and marginally significant campaign effects on these two outcomes. With respect to PrEP motivation, *post hoc* pairwise comparisons applying the Sidak adjustment for multiple comparisons revealed greater PrEP willingness among participants who viewed the *PrEPare for the Possibilities* campaign or the *PlaySure* campaign compared with the *PrEP4Love* campaign. With respect to PrEP behavior, no specific pairwise differences emerged from *post hoc* comparisons applying the Sidak adjustment.

#### Replication of survey analyses with full sample

We repeated the MANCOVAs examining the effects of couple composition and campaign on advertisement judgments at Time 1, PrEP stigma (Time 1), PrEP motivation (Times 1 and 2), and PrEP behavior (Time 2) using the full analytic sample (Time 1: *n* = 96; Time 2: *n* = 73). This full sample included participants who failed the attention/manipulation check and were initially excluded. The same pattern of significant results emerged with two exceptions. First, in the *post hoc* pairwise comparisons of advertisement judgments by couple composition, advertisements with heterosexual couples only were rated as significantly more memorable than those with no couples (*p* = .034), whereas this difference was only marginally significant in the restricted sample (*p* = .063). Second, in the MANCOVA examining conditional and interaction effects of couple composition and campaign on Time 2 PrEP motivation, the conditional effect of campaign on PrEP motivation was only marginally significant (*p* = .063), whereas the effect had been significant in the restricted sample (*p* = .046).

### Focus groups

#### Focus group sample overview

A total of 18 individuals participated across the four focus groups, nine (50.0%) of whom reported participating in the survey phase of the study. Three (16.7%) reported not knowing whether they had done so. Group sizes ranging from 3–5. **Table 1** displays participant characteristics. Participants ranged in age from 22–62 years (*M*[*SD*] = 34[10.3]). Most were non-Latinx/Hispanic (88.9%) and identified as gay (61.1%) or bisexual (38.9%). Most (88.2%) had heard of PrEP prior to the study, nearly half (47.1%) reported having ever used PrEP, and over a third (35.3%) reported current PrEP use.

#### Focus group themes and their relevance to survey findings

Focus group themes and exemplary quotes are presented in **Table 3.**

**Table 3:** Qualitative findings from four focus groups (*n* = 18).

Focus Group Themes	Key Points	Illustrative Quotes^a^
Targeted advertisements can be stigmatizing	Targeted advertisements alienate targeted group	•It’s geared towards Black men, for the most part, and that is–it comes across as bad, whether you have sex with dudes or not. It’s–it’s bad. It’s almost like racism. [Laughter.] It’s like you’re over here, and everyone else is over here. You’re not–you’re terrible. You guys are bad. We don’t want you mixing with our people, so we’re gonna stay over here, but we’re gonna go ahead and do that to kinda keep you safe. [P12, FG3, Before]
	Targeted advertisements associate targeted group with HIV	•If it’s solely for Black and African American people I would say that it, it, it definitely hits the nail on the head. However, I feel that, um, to certain people it can come across as insulting ’cause it’s almost like saying we are the ones who have the disease, like we’re the only ones with the problem. [P2, FG1, After]
		•It also kinda paints a–a picture, whether that was the intention or not, that this is something that like, um, uh, gay men of color are kind of like, uh, doomed to enter to like, uh, the reality of being HIV-positive. [P9, FG2, Before]
Targeted advertisements can have other adverse consequences (beyond stigma)	Targeted advertisements fuel conspiracy theories	•I think a lotta people when it comes to marketing probably think the best idea might be to market–really market towards, uh, same gender lovin’, uh, Black male or same gender lovin’ men of color, period. So that might be why you often see, um, a lotta Black men. But conversely, that can also, you know, build some conspiracies around the drug as far as, well why they always, uh, you know, buildin’–why they always marketin’ towards Black men, um, or Black gay men? Can you trust it. . . ’Cause even though, you know, a lot of us think–know it’s somethin’ that can help us, some, uh, think that it is somethin’ that’s out to get us as well. [P16, FG4, Before]
	Targeted advertisements miscommunicate the scope of the consumer market	•Do only gay people take this? Like, is it a gay drug?. . . That’s what it looks like. [P13, FG3, After]
Advertisements featuring a diversity of people are preferable	Imagery should be inclusive of people of different races, sexual orientations, body types, ages, etc.	•I would love to see a line of people standing up, um, every height, every age, every race, just standing there, and then it’s PrEP. That’s it. This is who it represents. It doesn’t just represent Black men. It represents everyone who is sexually active. This day and age, anyone can contract HIV, so I think it needs to be broadcast to every type of person. [P12, FG3, Before]
		•Like I said before, have a heterosexual, uh, White couple. Only because you–just ’cause you wanna show diversity maybe and a–and an Asian couple as well. You know, you just wanna have diversity because you wanna show that it is somethin’ that can benefit everybody. Uh, and right here, majority of the people appear to be of–of color, uh, Black or Hispanic. [P16, FG4, After]
	Diversifying will make the advertisements more relatable to more people	•People buy products that relate to them. So when they see images or whatnot with people who look like them or identify with them they’re gonna be more likely to purchase that or to find out more information about that product. . . Like, I, for me, I wasn’t really too dead set on getting into PrEP and then I found out more about it and I was looking at some of the ads and whatnot and so I kinda went into that. So, you know, a diversity of the different people or different races and ethnicities, that’s kind of the target focus for me. [P3, FG1, Before]
	Diverse representations of Black people and Black SMM specifically are important	•I think having some diversity matters, but like I think, uh, like I think representation matters more. . . Diverse representations of Black love and–and acknowledging that like this doesn’t just affect Black people. . . There are a lotta different kinds of Black people. And like that diversity matters just as much. [P10, FG2, After]
		•One of the problems that, um, we in the, uh, gay Black community. . . there’s not a lot of representation for masculine men. . . when I look at [refers to *PlaySure* Black SMM advertisement] and I look at [refers to *PrEPare for the Possibilities* Black SMM advertisement], I see what appears to be two masculine Black men, uh, which could be eye-catching as well. Um [clears throat] and–and I guess the–I mean, the same could be said for, uh, majority of [column with diverse advertisements] as well. So that’s just what–I think that’s–I think that could help out as well, to show that there is representation in–within, uh, the–if we’re gonna target the Black gay community, there’s representation there as well. [P16, FG4, After]
*Divergent participant viewpoints favoring targeted advertising (expressed by minority)*	*Targeting based on HIV epidemiology is reasonable*	*•I don’t think it would be stigmatizing to*, *uh*, *to focus on the gay community*. *I mean*, *it’s–it’s just a fact*. *You know*, *it would be silly to somehow think that just having some generic marketing campaign with*, *you know*, *some blonde*, *blue-eyed 20-year-old girl in the ad*, *who is probably way down on the totem pole when it comes to*, *uh*, *potentially becoming HIV positive*, *would be silly*. *[P8*, *FG2*, *Before]*
* *	*Resources should be prioritized for reaching those who need PrEP the most*	*•I think it makes sense [clears throat] to*, *uh*, *focus on the gay community*. *I mean*, *we’re still the–we still have the highest rate of HIV infections*, *so if you want–if you want an ad to reach the greatest number of potential customers*, *then that’s the way to go*. *I’m assuming there’s–there’s not unlimited resources to do full campaigns for every single person that is at risk for HIV*, *so I mean*, *if you have to–if you have to choose*, *if that’s–I mean*, *if that’s the case*, *then I think gay men*, *that’s probably the–the first group that you should advertise to*. *[P8*, *FG2*, *Before]*
* *	*Targeting can be received positively by targeted group*	*•I think that targeting communities actually lets [them] know that you care*. *Um*, *it actually lets [them] know that you have some cultural*, *like*, *competence about understanding them*. *[P2*, *FG1*, *Before]*
Advertisements without people are unappealing	•Images without people are not visually engaging	•[Referring to column of advertisements with no couples] In my opinion, if I were walking by and I see this advertisement at the train station, the, on those electric, um, screens, I wouldn’t bat an eye to it because it didn’t really catch my attention. [P5, FG1, After]
		•I don’t like [refers to column of advertisements with no couples] though … it’s boring. [P17, FG4, After]
*Divergent participant viewpoints favoring advertisements without people (expressed by minority)*	*Advertisements without people are a solution to targeting*	*•Advertisers can come up with more alternatives in–in who they choose for–for a particular ad for PrEP*.* *.* *. *worse come to worse*, *don’t put anybody*. *You know*, *like getting back to what I was saying*, *you know*, *use somethin’ else besides a person that look like they evidently*, *you know*, *slept around and*, *you know*, *and they’re gay*. *[P18*, *FG4*, *Before]*
* *	* *	*•I actually think–I actually think [refers to* PrEP4Love *advertisement with no couples]*, *I think that is the best ad in that whole*.* *.* *. *I like that more than anything else in that whole–in the rest of ’em*. *[Laughs] It just–it just doesn’t look–you know*, *they need somethin’ that*, *again*, *somethin’ that’s not targeting the average LGBT whoever*. *[P18*, *FG4*, *After]*
Highly sexualized advertisements can discourage PrEP	Sexualized imagery is unnecessary	•I don’t need to see intimacy. I don’t need to see kissing, hugging, touching, I don’t need to see skin. I think it–it’s more appealing when you’re watering the–the grass and just doin’ regular, everyday stuff. I think it kind of moving away from touching and sex. Um, they might get it and kind of explain that, hey, you can–I take PrEP, or I use PrEP or whatever, whatever the–the, um, text is. But I don’t necessarily wanna see anybody kissing or hugging. [P12, FG3, After]
		•I think I would show it as not bein’ somethin’ that is a medicine just for somethin’ sexual, if that makes sense. Kind of takin’–thinkin’ of it as somethin’ for like a vitamin, you know, just a, uh, it doesn’t have to be necessarily a sexual tie-in. . . So I just think that, to kind of take the trashiness out of it. [P16, FG4, Before]
	Sexualized images deter consumers who do not perceive themselves as highly sexual	•[Referring to advertisement in *PrEP4Love* campaign with diverse couples] Not a big fan of them sittin’ on top each other and, uh, so that’s pretty much the main one. And the–the same images larger [refers to Black SMM couple advertisement in *PrEP4Love* campaign]. Um, but some playfulness is okay, but. . . it also kinda depicts it as like, okay, well if you–like if you are a little more promiscuous and, you know, PrEP is for you. And if I’m not as promiscuous, maybe it’s not for me. [P6, FG2, After]
Context is paramount to the acceptability of sexual advertisements	Sexual advertisements belong in sexual spaces	•P9: In terms of like marketing, I like feel like these would all be in very different places, though. Like [refers to row of *PrEPare for Possibilities* advertisements], I feel like would be like on the metro bus or in the subway. And row–P10: In very mainstream places. P9: Yeah. And [refers to *PlaySure* row of advertisements], I feel like that would like be on like college campuses. And [refers to *PrEP4Love* advertisements], I feel like you might see like, you know, um, in like–P10: Online, at the club, at the bath house. [P9 and P10, FG2, After]
		•Especially on billboards and bus stops, you don’t wanna push sex and so onto my–tryin’ to have my sandwich on the train. . . and then I see this, and it’s disgustin’ whether it’s gay sex or straight sex. Doesn’t even matter. It’s just I don’t feel like I wanna see someone straddling anybody. I don’t wanna see her riding him [laughter] in the early mornin’. [P12, FG3, After]
		•It’s just a lot, um, that you almost would feel weird to stop and look at it. Like, that’s what I think is–like, if the bus stops, and I’m starin’ at this, and people are starin’ at me, [laughter] then I look, and–you know, like, what kinda moment would that be? [P11, FG3, After]
	Sexual advertisements are inappropriate for some viewers	•I think it just depends on the context of the advert as well, because as you were saying earlier, on dating apps, um, there is the assumption that people will be there looking for a sexual encounter. So I think that’s what they do, they’ll use, for instance, pictures that are in [refers to row of *PrEP4Love* advertisements]. Whereas, if it’s something on the Metro they might use something a bit less salacious that’s more family friendly just because they have to be mindful of the fact that there are more conservative people on the trains and the–you know, it’s a context where there are children and it’s not, you know, only sexual. [P2, FG1, After]
		•I don’t really care for it. I think it’s too sexual. . . [refers to diverse couples advertisement and Black SMM couple advertisement in *PrEP4Love* campaign] I think, uh, it’s just too sexual [laughter] for me. It’s a little much. So, if I’m ridin’, and I have my kid with me, I don’t–I don’t want him to see that. It’s kinda like soft porn. [P12, FG3, After]
Targeted advertisements that are sexual in nature can be especially stigmatizing	Targeting hypersexualizes Black people	•I think there’s somethin’ to be said, like, for maybe–sometimes with ads–and it’s harder to Black people. I feel like they gotta be like, super-explicit. But, like, think about ED commercials, where you got two people holdin’ hands in a bathtub. Like, there’s–there’s an ability to be suggestive. . . They’re not, like, all hugged up and, like, just dry-humpin’. Their significant other is, like, suggestive, like, oh, like, maybe handholding, maybe–I don’t know. I just think that, like, these come across like–to me, very, very, very, like, strong. [P11, FG3, After]
	Sexual advertisements of Black SMM can stigmatize Black SMM in mainstream public spaces	•If it’s going on a Jack’d ad or a Grindr, it should be sexual, ’cause you’re having sex, so I mean, like, I mean I–I mean I just think, like, that, it lends itself to a certain audience. So–If it was on a hookup app then that would be appropriate. If I am walking down and it’s next to Walgreens, I’m not sure that–I’m not sure if that is really the message that you’re–’cause then that can put out the stigma. Yeah, that would put out the stigma that that then Black gay men are suggestive and that’s all they are. [P3, FG1, After]

*Note*. Rows highlighted in blue reflect findings related to targeted advertising. Rows highlighted in yellow reflect findings related to the sexual nature of advertisements. Rows highlighted in green reflect findings related to the intersection of the two. Rows with italicized content contain viewpoints expressed by one or a few participants that diverged from the majority.

^a^Parenthetical content includes participant (P) number, focus group (FG) number, and whether the statement was made before or after the 4x3 grid of visual advertisements had been presented to the group.

SMM = Sexual minority men

Focus group findings largely corroborated survey findings. Many participants agreed that targeted advertisements were potentially stigmatizing. Participants explained that such advertisements alienated Black men and associated them with HIV. They further noted that excluding other demographics, such as White people and heterosexuals, propagated distrust and conspiracy theories about PrEP and failed to convey the full scope of the consumer market.

Consistent with survey findings indicating that advertisements featuring diverse couples were particularly motivating, relatable, and memorable, nearly all focus groups participants favored diverse advertisements. They expressed appreciation for the forms of diversity displayed in the diverse couples advertisements with respect to age, body type, gender, and masculinity. They recommended further diversifying the images by including a broader range of races, nationalities, and sexual orientations and underscored the value of diverse representations of Black SMM and other Black people. Participants acknowledged that diversifying increased the relatability of the advertisements for a wider range of potential consumers.

Just as a minority of participants rated Black SMM advertisements as “not at all stigmatizing” in the survey, a few focus group participants rejected the notion that targeting PrEP advertisements to Black SMM was stigmatizing. They justified a targeted approach from an epidemiological standpoint, mentioning the importance of prioritizing resources for the groups most affected by HIV. One participant also suggested that targeting a group could convey caring and cultural understanding, although this participant also vocalized support for advertisements featuring a diversity of couples.

Substantiating survey participants’ rating of the advertisements with no couples as being the least eye-catching, motivating, or memorable, participants largely disliked these advertisements, criticizing them as “boring,” and suggested the advertisements would be unlikely to capture their attention in public spaces. However, a few participants regarded them as more “family friendly” and desirable alternatives to advertisements with people.

Whereas minimal differences in advertisement judgments across campaigns emerged in the survey data, participants did express campaign preferences in the focus groups. The difference in sexual explicitness across campaigns was a central focal point, with the *PrEP4Love* campaign deemed the most explicit and the *PrEPare for the Possibilities* campaign deemed the least. Many participants objected to the highly sexualized nature of the *PrEP4Love* advertisements, particularly if the advertisements were to be displayed in public spaces (e.g., on the subway or on billboards) versus more overtly sexual spaces (e.g., on dating apps or in bath houses). Advertisements that targeted and sexualized Black SMM in public spaces were considered particularly prone to reinforcing stigma.

## Discussion

In the current study, we investigated Black SMM’s responses to targeted PrEP visual advertisements featuring Black SMM, including their judgments of the advertisements as well as the effects of the advertisements on PrEP stigma, motivation, and behavior compared with non-targeted advertisements. We found that many participants perceived targeted PrEP advertisements as stigmatizing and favored advertisements featuring greater diversity. With respect to the survey experiment, in the between-groups comparison of advertisements conducted at Time 1, there were significant effects of couple composition (targeting) but no campaign or interaction effects on advertisement judgments. Advertisements featuring Black SMM exclusively were perceived as more stigmatizing than advertisements featuring no couples. Advertisements with diverse couples—which included Black SMM as well as couples of other races and sexual orientations—were judged to be more eye-catching, motivating, relatable, and memorable than those with no couples. There were few couple composition, campaign, or interaction effects on PrEP stigma, motivation, or behavior. The within-subjects comparison of advertisement judgments at Time 2 yielded similar results to the between-groups comparison at Time 1, finding couple composition effects but limited campaign and interaction effects. Advertisements that featured Black SMM exclusively were again judged as more stigmatizing than advertisements featuring no couples, and advertisements with diverse couples were judged as more eye-catching and motivating than those with no couples. Focus group participants further corroborated concerns related to stigmatization and generally favored diverse and less sexualized advertisements, particularly for display in public spaces.

Our findings align with several recent qualitative studies in which Black SMM reported PrEP social marketing that targeted Black SMM or SMM more broadly to be stigmatizing and called for inclusion of greater diversity in visual advertisements [19, 23, 24]. Although ratings of the advertisements featuring a Black SMM couple exclusively did not significantly differ from ratings of the advertisements featuring diverse couples (including Black SMM) in our survey experiment, our focus group participants clearly distinguished between these two portrayals. They preferred the advertisements with diverse couples and perceived them as less stigmatizing than the advertisements with the Black SMM exclusively. As in prior studies, our participants recommended diversifying beyond Black SMM (e.g., including other sexual orientations and ethnicities in addition to Black SMM) and also diversifying representations of Black SMM (e.g., including Black people with different skin tones) [19, 23, 24]. Whereas prior studies took place in the Southern US, fostering speculation that these preferences may be geographically limited [24], our study suggests that Black SMM recruited in Washington, DC, and other East Coast cities also share this preference. Black and Latinx SMM in New York City have leveraged similar critiques of targeted social marketing with respect to HIV testing campaigns [36]. Thus, opposition to targeted HIV-related social marketing and a desire for greater inclusivity appears to be shared among SMM of color in multiple geographic locations, including relatively progressive urban areas.

Beyond reducing stigmatization, a more inclusive approach that represents Black SMM among a diversity of other races, sexual orientations, and genders could offer several other advantages. Including non-minoritized groups could be a strategic way of combatting the suspicion and conspiracy theories resulting from targeted PrEP advertising to Black SMM. By diffusing the association between PrEP and the sexual minority community, inclusive advertising could also enhance the accessibility of PrEP to Black SMM or others who wish to maintain privacy about their sexual behavior or identity [19]. Additionally, as focus group participants pointed out, more inclusive PrEP social marketing could broaden the reach of PrEP, conveying its relevance to a wider range of potential beneficiaries, including people at risk for HIV who do not belong to designated priority populations [14, 25].

Survey and focus group findings converged in their unfavorable evaluation of the advertisements with no couples. However, these findings must be interpreted with caution. The advertisements with no couples were created as a control condition for evaluating couple composition effects simply by removing the images of people in the original advertisements. Therefore, they depict the absence of people, not the presence of alternative imagery. Advertisements that lack people but include alternative imagery that is visually engaging may be more eye-catching and appealing, overcoming the weaknesses ascribed to the advertisements not featuring couples while retaining the key strength identified: low potential for stigmatizing or offending consumers.

PrEP social marketing initiatives have typically included people in advertisements and have widely embraced a sex-positive, pleasure-focused approach to health promotion [37]. Such initiatives reflect a growing literature documenting the beneficial effects of PrEP for people’s psychosexual wellbeing, including increasing sexual intimacy and options [38]. The three campaigns considered in this study all included imagery projecting flirtation, affection, or eroticism. As noted in our analysis of focus group participants’ response to textual elements of the PrEP advertisements, described in a separate article [34], many Black SMM in our study did not react favorably to the “sex-positive” aspects of the advertisements. Participants suggested that more sexualized advertisements may be better suited for sexual spaces, such as dating apps and bath houses, than spaces shared with members of the general public, including children.

Our findings suggest that sexualized imagery could amplify stigma by reinforcing sexual stereotypes ascribed to Black SMM [20]. However, it is important to contextualize these findings within the current era of advertising and visual media in the US, a time when Black SMM are rarely represented. The presence of Black SMM in a sexualized advertisement related to HIV may be perceived as especially stigmatizing in the absence of Black SMM represented elsewhere (e.g., in non-sexualized advertisements unrelated to HIV.)

Findings should also be contextualized according to our recruitment method. All of the recruitment flyers that we distributed featured a single Black man, and flyers varied by level of nudity. Some prospective participants—particularly those who perceived the flyer imagery to be stigmatizing—may have been deterred from participating, in which case, the strength of the association between targeted ads and perceived stigmatization may be an underestimate relative to Black SMM more broadly. Prospective participants who preferred advertisements with people (vs. alternate imagery) may have been more responsive to our study flyers, in which case, the less favorable attitudes toward the advertisements without couples may have been exaggerated in our sample relative to Black SMM more broadly.

We found that couple composition and campaign had minimal effects on PrEP stigma, motivation, and behavior. The only discernable effect was related to campaign, with participants who viewed the *PrEPare for the Possibilities* campaign or the *PlaySure* campaign reporting greater PrEP motivation eight weeks later compared with those who viewed the *PrEP4Love* campaign. A considerable proportion of participants reported seeking information and initiating PrEP between the Time 1 and Time 2 survey, but this was true across conditions. It is possible that viewing PrEP advertisements or participating in a study about PrEP more generally prompted such behavior change, but our study design prohibits such causal inference.

### Limitations

Our study has several limitations. First, there are limitations associated with the size of our sample. Despite our recruitment efforts, including incentives for participation, our survey and focus group samples were small. The size of the survey sample limited statistical power to detect significant effects. Underpowered studies, such as ours, are vulnerable to false positives and inflated effect sizes [39] as well as false negatives. Thus, the small sample size limits the generalizability of our survey findings, which should be interpreted accordingly. We recommend a more highly powered study be conducted in the future to establish the replicability of our findings. A larger sample would also allow for exploration of the potential moderating effects of sexual orientation (e.g., bisexual vs. gay), internalized stigma, advertisement viewing frequency, and other variables related to the participants or experimental manipulation.

Our four focus groups took place in Washington, DC, and encompassed 18 Black SMM altogether. The themes that emerged are not intended to generalize to US Black SMM broadly. For example, the reservations that participants vocalized around the sexual nature of some advertisements may not be shared by Black SMM and others who live in the communities where those advertisements were actually displayed.

Second, surveys were completed online and participant eligibility was based on self-report, conferring vulnerability to participant response bias and fraudulent responding. Although we implemented several measures to protect our survey and ensure the integrity of our data, we encountered several bouts of suspicious responding that necessitated interruptions in data collection and exclusion of survey records.

Third, we made several modifications to the PrEP advertisements used as visual stimuli that detract from the ecological validity of our findings (e.g., converting all images to black-and-white coloring, incorporating “+ condoms” into the *PrEP4Love* campaign). Additionally, the Black heterosexual couple depicted in the *PrEPare for the Possibilities* campaign was superimposed from a different PrEP campaign because the original *PrEPare for the Possibilities* campaign did not include any images of Black heterosexual couples. This fourth campaign, *StaySure*, was developed by the same institution as the *PlaySure* campaign (NYC Health Department), but the image did not overlap with *PlaySure* advertisement images.

Fourth, one of the three campaigns that we included, *PrEPare for the Possibilities*, was based in Washington, DC, where many survey participants were recruited and focus groups were held. However, as with the other campaigns, only a minority (23.3%) reported outside exposure to advertisements from this campaign before or during the study when surveyed at Time 2.

Fifth, the online research setting and circumstances under which participants viewed and evaluated the visual advertisements likely impacted participants’ viewing experience and consequent evaluation of advertisements. Focus group participants highlighted context as a key determinant of advertisement preference, which suggests that our findings may not represent the responses and preferences participants would have had if they had encountered the advertisements in everyday life. In particular, the deliberate, sequential viewing of advertisements during the within-subjects analysis (Time 2) or the prolonged, simultaneous viewing of advertisements during the focus groups—while advantageous for making systematic comparisons across advertisements—are unlike the ordinary viewing of a single advertisement in passing as part of daily life. The sequential and simultaneous viewing paradigms, whereby participants viewed images of multiple couples, may have attenuated the effect of viewing the Black SMM couple alone in an advertisement. However, the initial between-groups analysis would not have been subject to this particular limitation, highlighting the value of using multiple methods of assessment.

Finally, the measures we used have limitations. Several of our measures, including outcomes, were single-item measures rather than multi-item scales, which may reduce their predictive validity. Additionally, our measure of advertisement (study stimulus) viewing frequency represented the number of times the emailed advertisements were opened but did not account for the duration of exposure or viewer attention.

### Future directions

Further research is needed to investigate Black SMM’s differential judgments of advertisements featuring sexualized images of Black SMM vs. non-sexualized images of Black SMM, alone vs. among a diversity of other people. Such research could also systematically assess whether judgments of sexualized advertisements are impacted by viewing the advertisements in sexual vs. non-sexual spaces. Additionally, research examining Black SMM’s responses to advertisements featuring greater diversity than depicted in this study and advertisements that lack people but contain alternative objects of focus could help to optimize PrEP social marketing for Black SMM in the future.

Because of our focus on motivational and behavioral outcomes related to initial PrEP uptake, we limited the scope of the survey sample to include only individuals who had never used PrEP. Replication of the study with former PrEP users, whose advertising preferences and responses may vary systematically from PrEP-inexperienced individuals, could help to inform initiatives aimed at encouraging PrEP reengagement.

Future research could also adapt our study paradigm for use with other configurations of people (e.g., alone, in polyamorous relationships) and with other groups disproportionately affected by HIV (e.g., Latino SMM, Black cisgender and transgender women, people who inject drugs) to assess their response to targeted and non-targeted advertisements. If multiple priority populations favor images featuring diversity, this shared preference would bolster existing motivation for social marketing initiatives to embrace diversity in future advertising imagery.

## Conclusion

There is no one-size-fits-all solution to optimize the social marketing of PrEP to Black SMM. Black SMM are a heterogeneous group, and their perspectives and preferences related to PrEP social marketing are not monolithic. Nonetheless, a significant proportion of our participants regarded PrEP advertisements that targeted Black SMM to be potentially stigmatizing. Likewise, they expressed a preference for more diversity to be depicted in advertisements. Inclusion of both Black SMM and people with different sociodemographic characteristics within a single advertisement could honor this preference for diversity. By still including Black SMM, this approach could simultaneously ensure representation and fulfill the goal driving many targeted PrEP social marketing campaigns in the first place: to prioritize resources to a group disproportionally affected by HIV [8].

The sexual nature of the advertisements, particularly the most explicit of them, did not resonate with focus group participants in our study. Although sexual advertisements featuring Black SMM may have been designed to promote sex-positivity and combat stigma, participants in our study spoke to the potential for sexualized images of Black SMM to instead perpetuate stigma, reinforcing sexual stereotypes ascribed to Black SMM.

In an ideal world, stigma would not exist—in general, or relative to PrEP, HIV, and Black SMM specifically. Sex and sexuality would not be taboo topics, and imagery projecting Black SMM sexuality and encouraging PrEP use would be universally embraced. It is possible that increasing representation of Black SMM sexuality in media and advertising may help move us towards that ideal reality. However, in the present reality in which we live, where synergistic stigmas related to PrEP, HIV, and Black SMM persist, our study results suggest that highly sexualized images of Black SMM in PrEP advertisements may inadvertently reinforce stigma. Thus, looking ahead to the immediate future, if the primary goal of a PrEP social marketing campaign is to increase PrEP awareness and uptake, then our results suggest caution with respect to including highly sexualized images of Black SMM, particularly if the materials will feature Black SMM exclusively or will be displayed in public spaces. If instead the primary goal of the campaign is to defy antiquated boundaries and enhance mainstream representation of Black SMM sexuality, then incidental damages—including the advertisements failing to resonate with some Black SMM—may be necessary to accept in the pursuit of change.

## Supporting information

S1 Table Means, standard deviations, and bivariate correlations of all advertisement judgments (Time 1). (DOCX)

S2 Table Means, standard deviations, and bivariate correlations of PrEP stigma (Time 1), motivation (Times 1 and 2), and behavior outcomes (Time 2). (DOCX)
