# Supplementary material for: Targeted social marketing of PrEP and the stigmatization of black sexual minority men
Source: PLoS One. 2023 May 11;18(5):e0285329. doi: 10.1371/journal.pone.0285329 (PMC10174512; doi:10.1371/journal.pone.0285329)
Supplement: S1 Table — (DOCX) [file pone.0285329.s001.docx]

**S1 Table. Means, Standard Deviations, and Bivariate Correlations of All Advertisement Judgments at Time 1**

**
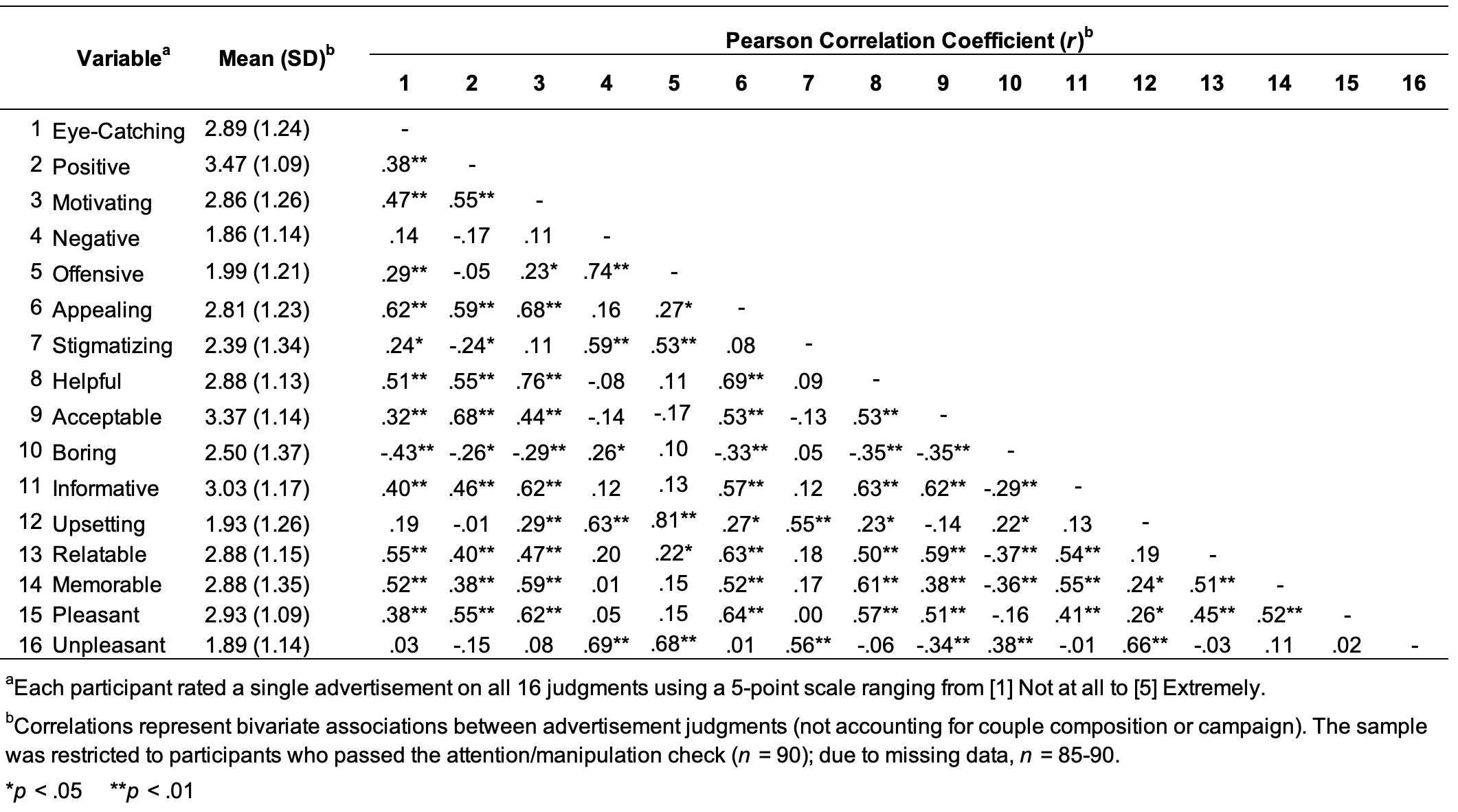
**
