# Supplementary material for: Targeted social marketing of PrEP and the stigmatization of black sexual minority men
Source: PLoS One. 2023 May 11;18(5):e0285329. doi: 10.1371/journal.pone.0285329 (PMC10174512; doi:10.1371/journal.pone.0285329)
Supplement: S2 Table — (DOCX) [file pone.0285329.s002.docx]

**S2 Table. Means, Standard Deviations, and Bivariate Correlations of PrEP Stigma, Motivation, and Behavior Outcomes**

**
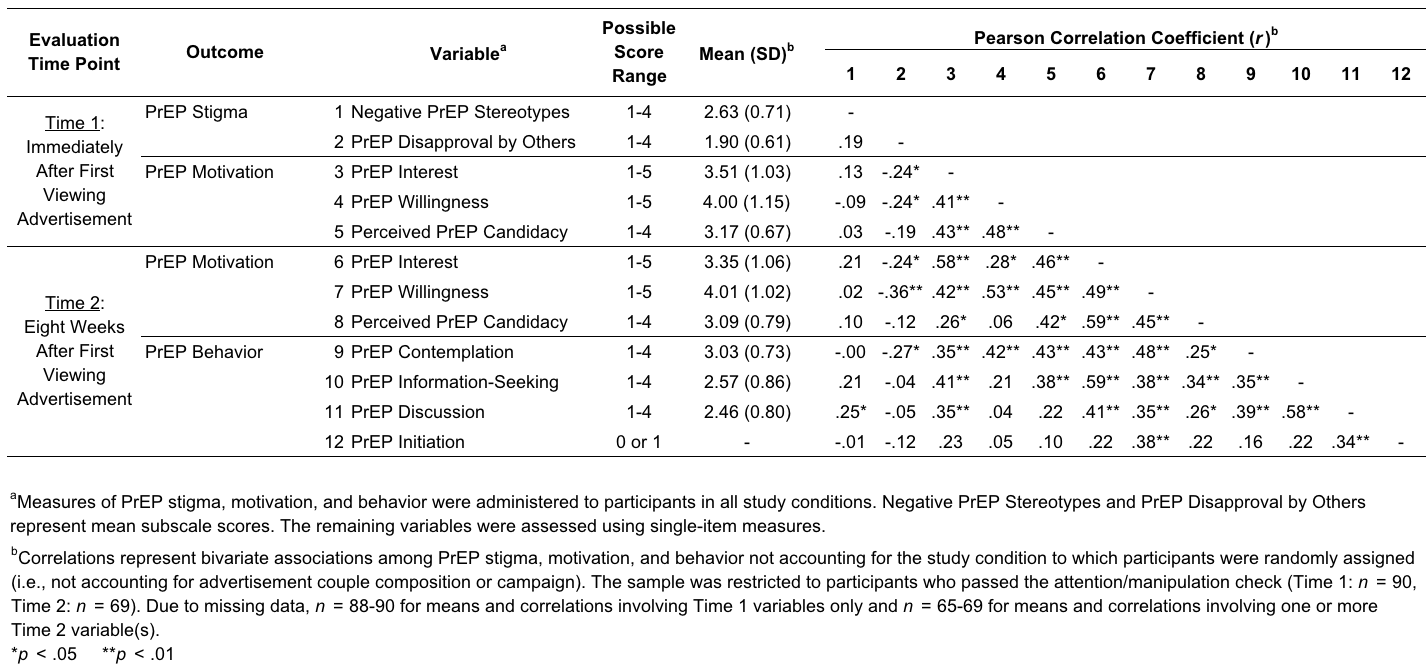
**
